# Cocaine in Eye, Ear and Throat Practice

**Published:** 1889-11

**Authors:** S. G. Dabney


### COCAINE IN EYE, EAR AND THROAT PRACTICE.

By S. G. Dabney, M. D.

It is certainly not too much to say that the province in which cocaine finds its most perfect application is the area of distribution of the fifth pair of cranial nerves. I wish to present briefly a few points in regard to its use in this region. The strength generally employed is two grains to the dram, or approximately four per cent. For operations on the eye it is best to instil a few drops of this solution about ten minutes before operating, and repeat in three or four minutes. Among the slight occular operations for which cocaine, so applied, is of the greatest service is the extraction of foreign bodies from the cornea; a drop or two of cocaine renders this otherwise acutely painful procedure altogether painless. The slitting up of the canaliculus for obstructed tear-passages is made much easier by the instillation of cocaine; the injection of the same into the lachrymal sac, and, if possible, through the duct, renders probing much less painful and sometimes allows the passage of a larger instrument. For this purpose I have also used a salve of cocaine and vaseline applied on the probe, but have found the previous injection of a solution more satisfactory. Cocaine renders iridectomy an almost painless procedure; the clipping of the iris does, however, still cause some pain. In the extraction or needling of cataract, local is in almost every case superior to general anesthesia. Among the disadvantages of chloroform or ether may be mentioned the vomiting which is apt to follow their administration, and its liability to cause prolapse of iris or vitreous humor, or other disaster to the eye. For entropion I have within the last two years operated several times according to a method recommended by Dr. Green, of St. Louis, in the American Journal of Ophthalmology; as this condition is due to a cicatrical contraction of the tarsal cartilage, causing incurving of the lid, the simplest procedure would seem to be to cut through the cartilage longitudinally from the mucous surface, and then for a short time to keep apart the surfaces of the cut so made by stitches passed under the skin of the lid and united over a little ball of cotton so as to thoroughly evert the lashes.

This is the course recommended by Dr. Green, and one I have found very satisfactory. Preparatory to this operation the lid should be turned out and a four per cent or stronger solution of cocaine applied, soaked in a pledget of cotton. There is apt to be quite profuse bleeding, and the hemostatic properties of cocaine come into excellent service. I have heard little in the last year or two of the tendency of cocaine to increase secondary hemorrhage; I have not observed such an effect myself. For the removal of chalazia, if the incision is to be through the skin, a few drops of a four per cent solution should be injected hypodermically. Here the lid-forceps of Desmarres or Knapp serve admirably, not only to restrain bleeding, but to prevent the diffusion of cocaine through the loose tissue of the lids to the general circulation.

In operations for strabismus cocaine is of great service, but the passage of the hook beneath the tendon and the cutting of the latter are sufficient to cause considerable pain, and, as this operation is frequently done in unruly children, a general anesthetic is often the best. In such cases I use chloroform. As a mydriatic preliminary to a thorough ophthalmoscopic examination, cocaine holds the first rank, not only because the mydriasis which it produces is of shorter duration than that from atropia or homotropia, but also because it is far less likely, in elderly people, to cause an increase of intra-occular tension. Cocaine does not suspend the accommodation sufficiently for it to be used in the examination of refractive errors, but is often serviceable to lessen the temporary injection and unpleasant effects of other mydriatics, and doubtless aids in completing the paralysis of the ciliary muscle which they induce. The photophobia of phlyctenular ophthalmia nearly disappears after the instillation of cocaine. Painful ulcers of the cornea may be rendered easy by this local anesthetic. For the pain of iritis, or other deep-seated inflammation, cocaine is of little or no avail.

Enucleation is the most important of operations about the eye for which cocaine is not suitable; it could only prevent pain in this operation when injected into the tissues of the orbit, and as it is impossible to restrict its diffusion from the cellular tissue here, such injection has been found decidedly dangerous, and most operators now use general anesthesia.

The disadvantages attending the use of cocaine in the eye are chiefly temporary mydriasis and paresis of accommodation it induces, a dessiccating influence on the corneal epithelium, and a follicular inflammation of the conjunctiva which follows its long-continued use.

Cocaine in the ear is of comparatively limited application; when the eustachian catheter is to be used, however, a spray of cocaine in the nose renders its passage far less disagreeable to the patient and easier to the operator. In inflammation of the ear, whether in the auditory canal only, or as is much more often the case involving the tympanum and parts adjacent, cocaine is of little or no service.

In the nose, more than in any other part of the body, we see the wonderful influence of cocaine in exciting the vasso-constrictor nerves and thus lessening the caliber of the arterioles. When the erectile tissue covering the turbinated bones is distended, perhaps even to touching the septum, the application of cocaine produces such contraction as in a few minutes to open the nose most completely. This relief from nasal obstruction, due to distention of erectile tissue, lasts a variable time, not usually more than an hour or so. The small cocaine atomizer sold in the drug stores furnishes an excellent means for its application. The patient should be warned not to snuff back the spray, as cocaine in the naso-pharynx produces a very disagreeable sensation of a foreign substance there. Lately the representative of a prominent wholesale druggist left me a sample of what was claimed to be tasteless cocaine, but two patients upon whose throat I have used it, complained as much as is usual of its very bitter taste. The anesthesia produced by cocaine in the nose is sufficient for nearly all of the many nasal operations which have of late come into vogue. Before using the knife or saw for deflected or thickened septum, or the galvano-cautery, I find it best to apply the cocaine, preferably a ten per cent solution soaked in a little pledget of cotton, and allowed to remain in the nostril for about five minutes. In several cases I have seen constitutional eflects from a four per cent solution of cocaine used in the nose, but they have never been at all alarming; usually there is first a slight exhilaration, followed by decided depression. but occasionally the first effect of the drug, following a few minutes after its application, is a feeling of faintness and nausea.

One peculiar effect of cocaine on the throat should be mentioned, namely, temporary difficulty in deglutition. This has been accounted for by some on the supposition of a paresis of the muscles of the throat, while by others it is attributed to a partial suspension of the reflexes frum the local anesthesia. An incautious attempt to swallow in such cases may allow the food to pass into the windpipe and excite symptoms of an alarming appearance for a few minutes.

Of cocaine in the pharynx and larynx there is also much to be said; but, as I wish to make my paper brief, I will recall only a few of its special indications.

The little operation of clipping the uvulva may be done without the least pain and with only very trivial bleeding. The removal of a part of the tonsils is rendered much easier if a solution of cocaine has been painted over the surface. Localized soreness and pain on deglutition may be greatly relieved by the applications of a solution of cocaine in certain cases, but only when the inflammation is superficial. I have tried cocaine lozenges, put up by local manufacturing druggists, but have had no result from them.

In tubercular laryngitis cocaine does more in my experience to lessen the pain of deglutition, and thus to prolong life, than any other agent we possess. In such cases a spray of a four per cent solution is very effective.—*A. P. and N.*

